# Body Size, Extinction Risk and Knowledge Bias in New World Snakes

**DOI:** 10.1371/journal.pone.0113429

**Published:** 2014-11-19

**Authors:** Bruno Vilela, Fabricio Villalobos, Miguel Ángel Rodríguez, Levi Carina Terribile

**Affiliations:** 1 Departamento de Ecologia, Instituto de Ciências Biológicas, Universidade Federal de Goiás, Goiânia, Goiás, Brazil; 2 Departamento de Ciencias de la Vida, Universidad de Alcalá, Madrid, Spain; 3 Laboratório de Macroecologia, Universidade Federal de Goiás – Campus Jataí, Jataí, Goiás, Brazil; INIBIOMA (Universidad Nacional del Comahue-CONICET), Argentina

## Abstract

Extinction risk and body size have been found to be related in various vertebrate groups, with larger species being more at risk than smaller ones. We checked whether this was also the case for snakes by investigating extinction risk–body size relationships in the New World's Colubroidea species. We used the IUCN Red List risk categories to assign each species to one of two broad levels of threat (Threatened and Non-Threatened) or to identify it as either Data Deficient or Not-Evaluated by the IUCN. We also included the year of description of each species in our analysis as this could affect the level of threat assigned to it (earlier described species had more time to gather information about them, which might have facilitated their evaluation). Also, species detectability could be a function of body size, with larger species tending to be described earlier, which could have an impact in extinction risk–body size relationships. We found a negative relationship between body size and description year, with large-bodied species being described earlier. Description year also varied among risk categories, with Non-Threatened species being described earlier than Threatened species and both species groups earlier than Data Deficient species. On average, Data Deficient species also presented smaller body sizes, while no size differences were detected between Threatened and Non-Threatened species. So it seems that smaller body sizes are related with species detectability, thus potentially affecting both when a species is described (smaller species tend to be described more recently) as well as the amount of information gathered about it (Data Deficient species tend to be smaller). Our data also indicated that if Data Deficient species were to be categorized as Threatened in the future, snake body size and extinction risk would be negatively related, contrasting with the opposite pattern commonly observed in other vertebrate groups.

## Introduction

A general attribute that can be easily measured in every species on earth is its body size. Understanding how to make ecological predictions based on body size remains an important research goal in ecology since, in general, it is easier to measure morphological traits than to directly assess ecological aspects [1], [2]. For instance, a major question is whether ecological features correlated with body size can render any size class more or less susceptible to extinction. In other words, are small- or large-bodied species (or any size class in between) more vulnerable to extinction?

Analyses based on species' conservation statuses, such as those included in the IUCN Red List of Threatened Species [3], have shown that Threatened vertebrate species tend to be larger than Non-Threatened ones, at least for mammals [4], [5], birds [6], [7], frogs [8], and marine fishes [9]. Therefore, some ecological implications of body size may be causing large-bodied species to become more vulnerable to extinction. For instance, large-bodied species tend to have lower local abundances and, hence, are more likely to suffer from endogamy and loss of genetic diversity from stochastic processes [10]. Also, larger species are often more prone to be targeted by human hunters, fishermen or for general commercialization [9], [11], [12], or to suffer from habitat loss [4] owing to their greater energetic demands and their consequent need for larger home ranges [13]. Finally, large-bodied species typically reproduce at lower rates and have smaller litters than small-bodied species, thus being less able to recover from population decline [8], [14].

Generalizing the above explanations to all taxa is challenging owing to different or unknown body size-ecology relationships in poorly known taxa. For example, one could argue that large body size does not necessarily imply higher energetic demands because metabolic rate also depends on behavior [15], or that large-bodied species do not always show low local abundances [e.g. 16]. Additionally, extinction risk related to large body size may be undermined by other species' traits conveying less vulnerability to local threats, such as large geographic ranges and high dispersal capability [17]. Moreover, exceptions to the large species–higher vulnerability pattern have been found, for example, in Australian Elapidae snakes [18], freshwater fishes [9], and fossil bivalves [19].

Another caveat in generalizing the large species-vulnerability pattern is its dependence on conservation status assessments based on available information. Red Lists provide summarized information on species' threats, population trends, and conservation status, but are not free of biases as they depend on data availability [20], [21]. Thus, it is possible that small-bodied species are poorly studied owing to their more cryptic nature, narrow ranges and less charismatic appeal. Indeed, such characteristics of small-bodied species make them hard to discover and thus tend to be scientifically described more recently [22], resulting in less time for their study and, possibly, for accumulating relevant information to establish their level of threat. Overall, this could result in more small-sized species being assigned a conservation status related to uncertainty (i.e. categorized as Data Deficient) or not even being evaluated in comparison with larger species, which means that conclusions about the large species-vulnerability pattern could be misleading when based solely on species categorized as Threatened or Non-Threatened.

Here we focus on the New World's snakes (superfamily Colubroidea) for which we first asked (1) how species distribute within major IUCN extinction risk categories across countries and (2) whether species' description years – and consequently the time that has been available to accumulate biological information about them – are related with these categories and with body size. We also tested (3) if the large species-higher vulnerability pattern holds for these snakes, and (4) explored the extent by which the existence of species categorized as Data Deficient (i.e. with inadequate information on their distributions and/or population statuses to assess their risk of extinction [3]) and Not-Evaluated (i.e. that are awaiting evaluation against the criteria [3]) could affect the existence of such pattern.

## Materials and Methods

### The data

We generated a checklist of all extant New World snakes (superfamily Colubroidea) using the Reptile Database [23]. The final list comprised 1,240 species, their description years, and the countries where they occur; that is, 23 countries plus the Insular Caribbean countries that we treated as a single geographical unit (hereafter Caribbean Region) owing to their smaller sizes and poorer snake faunas. Conservation statuses of snakes were obtained from the IUCN Red List of Threatened Species [24], with a total of 456 species in our database (36.8%) being included in extinction risk categories. We could not use the finer IUCN classification scheme owing to low species numbers in some categories. Therefore, we employed two broader risk categories classifying species as either Threatened (TE; 4.4%) or Non-Threatened (NT; 25%), with the former including three finer IUCN risk levels (Critically Endangered, Endangered and Vulnerable) and the latter two (Near-Threatened and Least Concern). We also considered two additional categories comprising those species classified as Data Deficient (DD; 7.4%) by the IUCN, as well as species not included in the Red List but present in our database, which we termed as Not-Evaluated (NE; 63.2%).

We obtained Maximum Total Lengths in millimeters – a commonly reported body size measure for snakes [e.g. 25]– for 665 species in our database (53.6%) from Terribile et al. [26] for viperids and elapids, and from different sources for colubrids [27]–[30] (see details in Table S1). We log_10_-transformed all length measures prior to analysis [e.g. 31].

### Numerical methods

Our sample was not obtained following a random design but instead comprised the available data. This could have resulted in a taxonomically biased database unsuitable to generate robust conclusions regarding New World snakes. We thus first checked whether our data were equally distributed among families, subfamilies and genera by computing the taxonomic distinctiveness metric and comparing it with a random distribution [see 32, 33]. We found that our sample was more representative (i.e. contained more supraspecific taxa) than expected by chance (Δ+ = 79.445; p<0.001). Hence, we concluded that severe taxonomic biases were unlikely in our data.

Different species share different proportions of evolutionary history; thus whenever species are the sampling units in parametric statistical analysis (see below), the assumption of independence among observations cannot always be assumed (i.e. the closer the species in the phylogeny the more similar their characteristics might be [34]). Therefore, if phylogenetic autocorrelation were to be found in species' description years, body sizes, or both, this should be taken into account in the analyses. To evaluate the presence of phylogenetic autocorrelation in these variables, we used the most comprehensive phylogenetic hypothesis for reptiles available until now [35] that comprises 288 (43.3%) species in our database. We completed this phylogeny with the remaining species whose genera were already represented in the phylogeny (271, 40.8%) including them as polytomies; whereas we excluded the remaining species (106, 15.9%) from this analysis.

Subsequently, we generated phylogenetic global Moran's *I*s and Moran's *I* correlograms (in this case using five phylogenetic distance classes with equal number of observations [36]) for both description years and body sizes to asses levels of phylogenetic autocorrelation in these variables. We found virtually no phylogenetic autocorrelation in description years, neither globally (global Moran's *I* = 0.05; p<0.001) nor in any distance class (Fig. S1), indicating that there is no need to control for phylogenetic relatedness in analyses involving this variable. In contrast, we found significant phylogenetic autocorrelation for body size (global Moran's *I* = 0.19; p<0.001), particularly in the first distance class (Fig. S2), indicating that closely phylogenetically related species tend to have more similar body sizes.

Additionally, we also checked whether these lacks of phylogenetic autocorrelation in description years as well as the phylogenetic dependence observed for body sizes were robust to potential effects introduced by existing polytomies in our phylogeny (all of them circumscribed to the genus level). We did this by randomly resolving the polytomies 1000 times and computing the abovementioned Moran's *Is* for each run. No substantial changes were observed in any case (mean global Moran's *Is* ±SD were 0.048±0.003 for description year, and 0.187±0.006 for body size). This indeed supported our previously stated conclusion that while closely related species do not show a phylogenetically autocorrelated pattern regarding their year of description, they do show it when it comes to body sizes (more closely related species tend to show more similar sizes). Based on this, we used Phylogenetic eigenVector Regression (PVR) to generate a set of variables (phylogenetic eigenvectors) representing the phylogenetic relationships among snake species, and then regressed body size against these variables using an iterative search for the subset of eigenvectors that reduce the largest amount of autocorrelation in regression residuals [see 37]. Two eigenvectors (first and third) were selected for this procedure, which we used as covariables accounting for body size phylogenetic autocorrelation in our statistical analyses (see below), thus guaranteeing the statistical assumption of data independence [37].

We used one-way ANOVA to investigate differences in species' description years among risk categories and, if found, we applied planned comparison tests to evaluate a potentially structured sequence of risk categories according to species' description years. Specifically, we asked (1) if Non-Threatened species — which are expected to be abundant and, hence, easier to find — were described earlier than Threatened ones (description year for NT>TE); and if species in these two groups were described earlier than either (2) Data Deficient (TE+NT>DD) or (3) Not-Evaluated species (TE+NT>NE).

Regarding snake body size, we used multiple regression analysis to investigate relationships between species' body size and description years, and then used one-way ANCOVA to check for differences in body size among risk categories. We also applied planned comparison tests to investigate (1) if Threatened species were larger than Non-Threatened (TE>NT), and if species in these two groups were larger than either (2) Data Deficient (TE+NT>DD) or (3) Not-Evaluated (TE+NT>NE) species.

Mean sizes of Threatened and Non-Threatened species may change in the future owing to potential designation of Data Deficient and Not-Evaluated species within these risk categories. In order to explore these possibilities, we generated two extreme scenarios: that all Data Deficient or Not-Evaluated species end up being classified as either (4) Threatened (i.e. TE←{DD or NE}>NT) or (5) Non-Threatened (i.e. TE>NT←{DD or NE}). Although not realistic, these two extreme scenarios represent the largest effects that could be expected for the future changes on the large body-extinction risk pattern as a result of including poorly known groups within a risk category. In all the analyses involving body size, we used the two selected phylogenetic eigenvectors previously described as covariables to account for the phylogenetic autocorrelation existing in this variable (see above).

For all analyses, p-values were computed using simple F-tests as well as a randomization protocol that generated null distributions of body sizes and description years by randomly reshuffling these data across species 1,000 times. For each of these subsamples we obtained the F value of the corresponding analysis (i.e. ANOVA or regression), and the resulting 1,000 values were compared with the empirical F value. P-value results thus generated are expected to be more robust against potential biases in data [38], but in our case these were qualitatively similar to the ones given by classical F-tests, and we only reported the latter for simplicity. All analyses were performed in R 3.1.0 [39], using the packages ape [40], phytools [41] and letsR [42].

## Results

### Snake richness, extinction risks and geographic patterns

**Figure 1:**
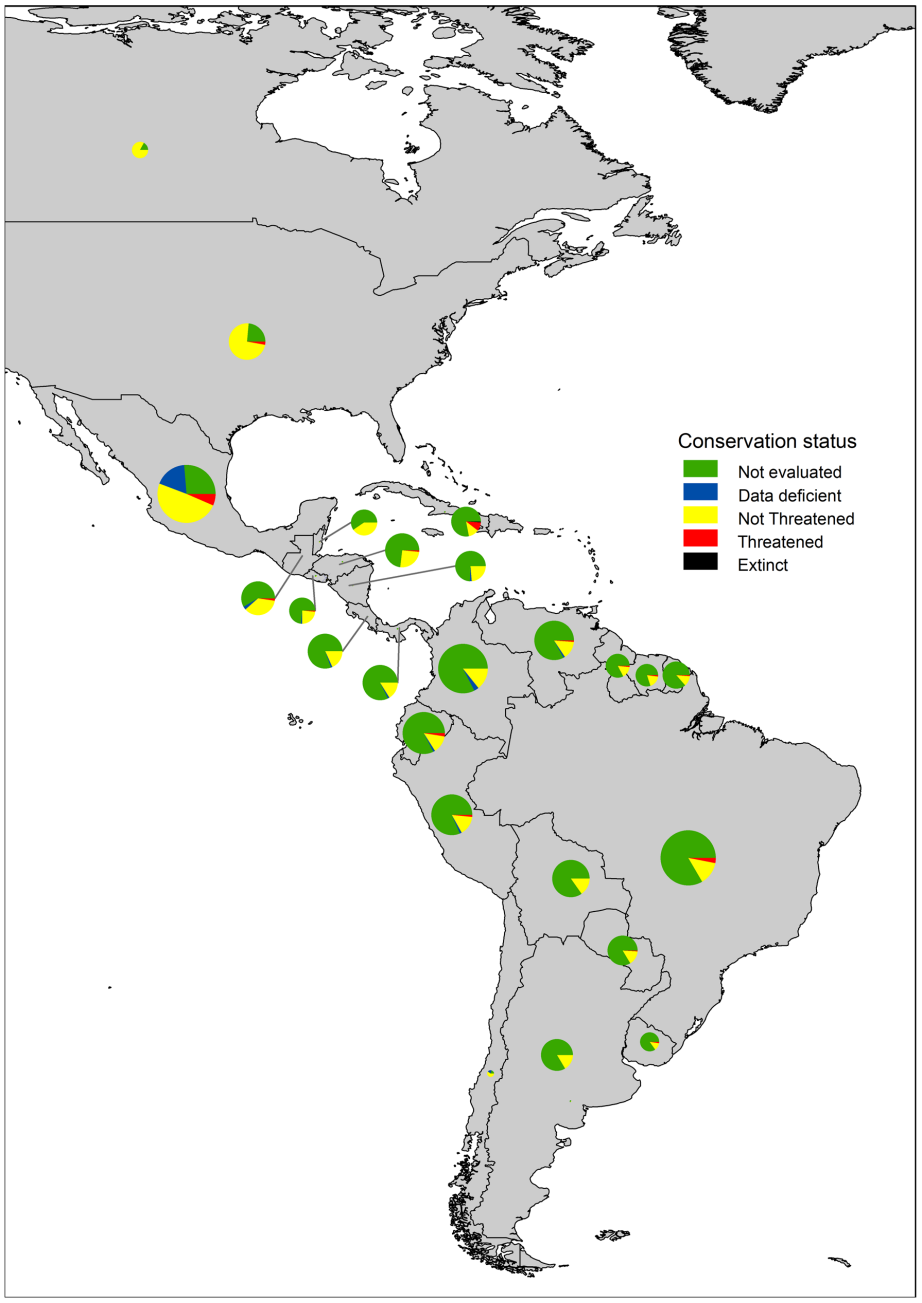
Country proportions of New World Colubroidea snake species in four conservation status categories: TE: Threatened, NT: Non-Threatened, DD: Data Deficient and NE: Not-Evaluated. For each country, circle size is proportional to the number of species it contains (note that the circle for Chile is hard to observe owing to its very low number of species (5) compared to the other countries).

The IUCN evaluation of the conservation status of Colubroidea species across the New World is far from complete (Fig. 1). This is less dramatic for North American countries (Canada, USA and Mexico) and Chile (<30% of their species await evaluation) than for the other countries, which exhibited between 58% and 87% Not-Evaluated species. Mexico holds the richest Colubroidea snake fauna in the New World (368 species, 29.7%) but also the largest number of species in the Threatened (26.1%) and Data Deficient (71.7%) categories, despite its relatively higher evaluation completeness. These patterns convey on Mexico a strategic importance for Colubroidea conservation. Brazil (334 species) and Colombia (268 species) are also species-rich, and Brazil and the Caribbean Region rated second and third in Threatened species numbers (10 and 9, respectively), so these countries also deserve special attention.

### Risk categories and species' description years

**Figure 2:**
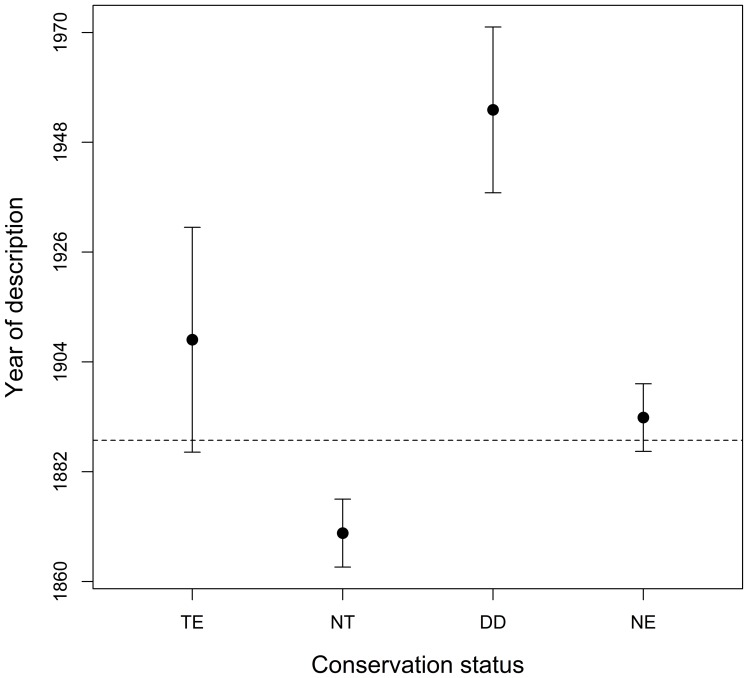
Description year comparison among four conservation status categories (TE: Threatened, NT: Non-Threatened, DD: Data Deficient and NE: Not-Evaluated) of New World Colubroidea snakes. Means and 95% Confidence Intervals are presented. Dashed line represents total mean. Note that the Y axis was drawn in log scale.

We found significant differences in species' description years among extinction risk categories (F_3;661_ = 20.65, p<0.001; Fig. 2), and in two of our planned comparison tests: NT>TE (F = 3.222; p = 0.001) and TE+NT>DD (F = −5.169; p<0.001), but not in the third: TE+NE>NE (F = −0.557; p = 0.577). This suggests a relationship between extinction risk and the temporal sequence of species descriptions in which Non-Threatened species tend to be described earlier (23 years on average) than Threatened species, and species in these two groups earlier (55 years) than Data Deficient species, but not earlier than Not-Evaluated species.

### Snake body size and extinction risk categories

**Figure 3:**
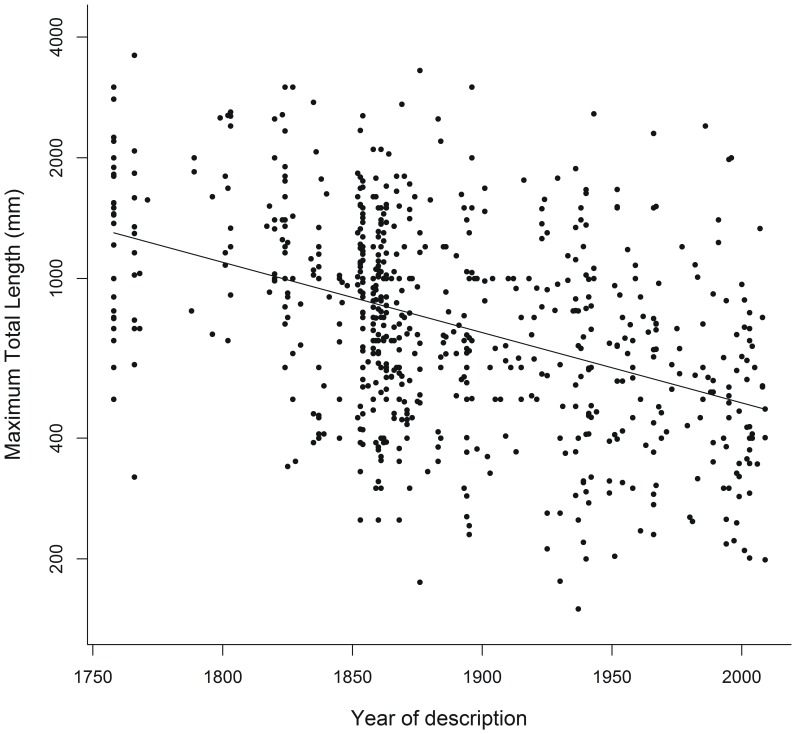
Relationship between description year and body size (measured as log_10_ maximum total body length) in New World Colubroidea snakes. Note that the Y axis was drawn in log scale.

**Figure 4:**
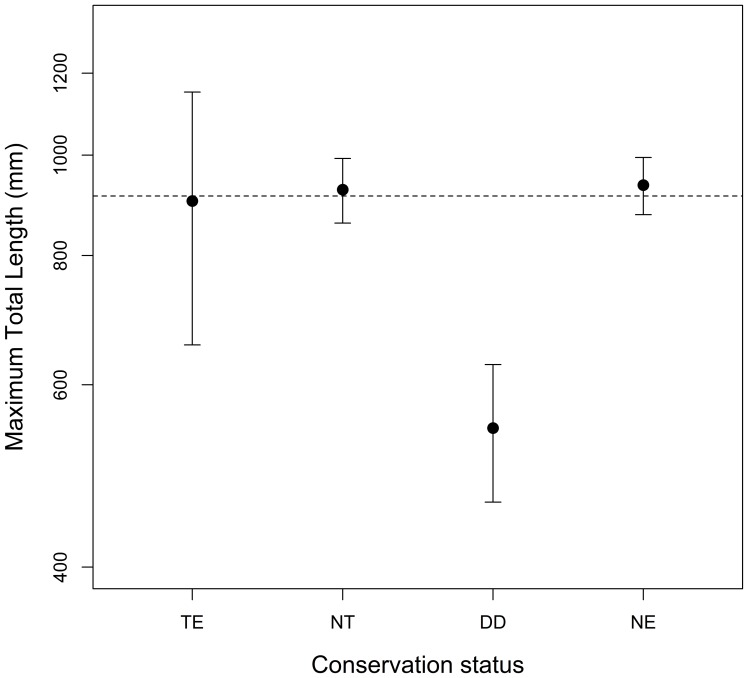
Mean body size (log_10_ maximum total body length) values of Colubroidea snake species in four conservation status categories (TE: Threatened, NT: Non-Threatened, DD: Data Deficient and NE: Not-Evaluated) across the New World. Error bars are for 95% Confidence Intervals; dashed lines represent total means.

Mean body length of New World snakes was 913±21.6 mm, ranging from 150 mm for *Tantillita brevissima* to 3600 mm for *Lachesis muta*. Regressing species body length against description year revealed a weak, but statistically significant negative relationship (r = −0.53; d.f. = 599; p<0.001; Fig. 3). That is, recently described species tend to be shorter than species described in previous years. Furthermore, significant differences were detected for body lengths across risk categories (F3;597 = 6.02, p<0.001; Fig. 4), and for the planned comparison checking for smaller body size in DD species (i.e. TE+NT>DD, F = 3.575, p<0.001), but not for the other comparisons tested (TE+NT>NE: F = 0.015, p = 0.987, and TE>NT: F = −0.334, p = 0.738). In fact, the latter comparison would still remain non-significant even if all DD species were to be classified as NT in the future (TE>NT←DD; F_2; 597_ = 0.015; p = 0.901); whereas the same comparison would become significant if all DD species were to be classified as TE (TE←DD>NT; F_2; 597_ = 9.377, p = 0.002). That is, Threatened species would then be considered shorter, on average, than Non-Threatened species. Conversely, potential future evaluation of Not-Evaluated species seems unlikely to alter the mean sizes of Threatened and Non-Threatened species, as indicated by the lack of support found for the corresponding simulated scenarios (TE← NE>NT; F_2;598_ = 0.195, p = 0.659 and TE>NT←NE; F_2; 598_ = 0.0535, p = 0.818).

We obtained similar results between the New World data and data based on the Mexican species only. There were significant differences in mean body length across risk categories (F_3; 231_ = 10.63, p<0.001), and the only significant planned comparisons were TE+NT>DD and TE←DD>NT (F = 3.322, p<0.001; and F = 8.525, p = 0.003, respectively). Therefore, differences across risk categories found using all New World data were not an artifact caused by the high concentration of Data Deficient species in Mexico (Fig. 1).

In sum, the data indicate that Data Deficient species were, on average, 358 and 381 mm shorter than Threatened and Non-Threatened species, respectively, and no differences in body length were detected either between Threatened and Non-Threatened snakes (although this could change in the future if current DD species were classified as TE), nor between these groups and Not-Evaluated snakes.

## Discussion

Previous work on mammals [4], [5], birds [6], [7], frogs [8] and marine fishes [9] found a positive relationship between body size and extinction risk. However, our results for New World Colubroidea snakes not only did not find this large species-higher vulnerability pattern, but indicated that if all species currently considered as Data Deficient were to be classified as Threatened in the future, the pattern would actually be the reverse (i.e. smaller species would be, on average, more at risk than larger species).

Body size has been commonly related with other biological traits affecting species' vulnerability to threats [4], [8]. For example, in endotherms, body size is often correlated with metabolic rate [43], and higher metabolic rates often mean increased food intakes, hence larger home ranges, which in turn may lead to higher susceptibility to habitat loss, the main contemporary threat to biodiversity [44]. However, energetic demands in ectotherms are typically much lower than in endotherms [45]. Therefore, even if correlated with body size, metabolic rate in ectotherms may not vary drastically among species of different body size and even large-bodied species could potentially survive in small areas after habitat loss, if enough food and microhabitat remain available [43]. Thus, we may expect the impact of habitat loss to be detrimental for large-bodied snakes but not as strong for large-bodied endotherms.

Species' geographic range size has also been related to extinction risk [46], [47] and it is usually assumed that large ranges confer less vulnerability to extinction as local threats are unlikely to strongly affect species' total distributions [47]. In snakes, range size is also positively correlated with body size [25], [48]. Additionally, low reproductive rates have also been associated with increased vulnerability to extinction [8], [14]. However, in contrast to other vertebrates where reproductive rate correlates negatively with body size, large-bodied snakes often have higher reproductive rates than smaller snakes [49]. Higher reproductive rates may confer higher fitness to species, increasing population abundance and facilitating recovery from disturbance, making species less vulnerable to stochastic processes such as genetic drift and endogamy [10]. These aspects (i.e. the positive relationships of snake body size with range size and reproductive rate) may, at least partially, explain the lack of relationship between body size and extinction risk found in the present study.

Also, compared to mammal species, snakes present a narrower range of body size variation that could minimize differences between large and small species related to threat vulnerability. Additionally, unlike birds and mammals, commercial exploitation of snakes is usually linked to traits not strictly related to body size, such as magic rituals and skin commerce [50], [51]. Thus, we could expect that large-bodied snakes are not necessarily more prone to hunting like it is for mammals and fishes [4], [9]. Therefore, large and small snake species may be similarly affected by threats, hindering the observation of a positive relationship between body size and extinction risk.

Beyond body size, other traits may better predict snakes' conservation status. For instance, Reed & Shine [18] found that behavioral characteristics are more important than body size in determining the conservation status of Australian Elapidae snakes. They suggested that sit-and-wait foragers are more likely to be threatened than active predators, mainly because the former depend on specific habitats that are more susceptible to anthropogenic activities. Filippi & Luiselli [52] also found that some life-history traits (e.g. synchronized mating season) increased the risk of extinction of Italian snakes. These studies highlight the need to include other species' traits, such as behavioral characteristics, to determine the relationship between different traits and extinction risk in snakes.

The year of description may affect the definition of species conservation status and related traits. For example, species described a long time ago have had more time to be studied and, consequently, more populations may have been discovered, enabling their categorization as less vulnerable or Non-Threatened. In our analyses, the year of description varied among risk categories. Non-Threatened species had the lowest mean description year, suggesting they had been easier to find, which is likely related to larger geographic ranges and/or abundances, two common characteristics of less vulnerable species. Thus, description year may determine the amount of available information, which in turn influences the categorization process. Accordingly, species classified as Non-Threatened have been described earlier, followed by Threatened species, and later by Data Deficient species. Further research on other taxa may establish the generality of the temporal pattern of description year among risk categories across vertebrate groups. Finally, unlike Data Deficient species, Not-Evaluated species did not show description year differences with either Threatened or Non-Threatened species; hence future risk categorization of species in this group are unlikely to modify our observed patterns.

To our knowledge, this is the first study relating vertebrate body size and extinction risk that has included Data Deficient species and, interestingly, we found that these species have, on average, smaller body size than species in categories with ‘adequate data’ (i.e. Threatened and Non-Threatened snakes). This finding suggests that small-bodied snake species have been poorly studied compared to large-bodied species. Such interpretation is consistent with our finding of a negative relationship between body size and description year, with a similar relationship found by Reed & Boback [53] for Australian and North American snakes, and with our observation that, on average, Data Deficient species have been the ones described more recently. New snake species are rarely encountered [54], possibly because snakes are typically hard to find in the field owing to their low activity rate, cryptic nature, and solitary habits. Our results clearly suggest that these challenges might be greater for smaller snakes, which in turn could be affecting the evaluation of the conservation status of species in this size class.

In summary, our data showed that the large species-higher vulnerability pattern present in different vertebrate taxa could not be generalized to New World snakes and suggests that care should be taken when trying to describe relationships between species traits and conservation status. For instance, comparing the body size between Threatened and Non-Threatened species may hide the influence of poorly-studied species in determining observed patterns. Future studies should consider these possibilities, analyzing all species and taking care of potential biases in order to clearly understand what makes species more or less vulnerable to extinction and contribute to their conservation.

## Supporting Information

Figure S1 (A) Phylogenetic Moran's I correlogram for description year in New World snakes. The analysis was done at five phylogenetic distance classes including equal numbers of species. (B) Description years (bars) structured into the phylogenetic tree. Colors represent the categories of threat used in the study; red: Threatened; green: Non-Threatened; blue: Data Deficient; and gray: Not-Evaluated.
(TIF)

Figure S2 (A) Phylogenetic Moran's I correlogram for observed body sizes (dots) and residuals after phylogenetic control (squares) for New World snakes. The analysis was done at five phylogenetic distance classes including equal numbers of species. (B) Body size (bars) structured into the phylogenetic tree. Colors represent the categories of threat used in the study; red: Threatened; green: Non-Threatened; blue: Data Deficient; and gray: Not-Evaluated.
(TIF)

Table S1 **Species IUCN red list conservation status (Following broader risk categories: TE = Threatened; NT = Non-Threatened; DD = Data Deficient; NE = Not-Evaluated) in 2012, maximum total length in millimeters and data source.**
(DOCX)
