# Supplementary figures and images for: Body Size, Extinction Risk and Knowledge Bias in New World Snakes
Source: PLoS One. 2014 Nov 19;9(11):e113429. doi: 10.1371/journal.pone.0113429 (PMC4237443; doi:10.1371/journal.pone.0113429)

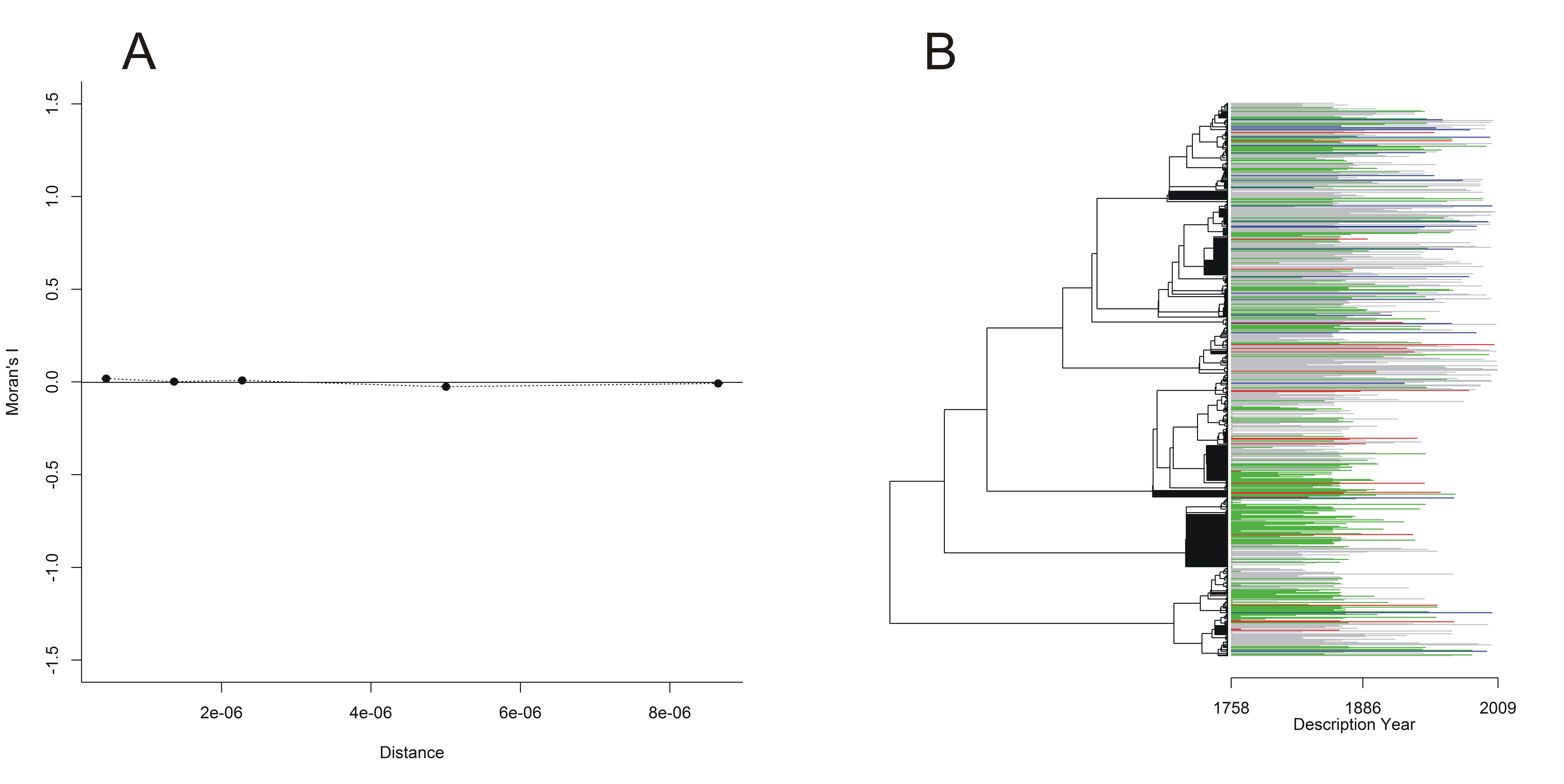

Supplement: Figure S1 — (A) Phylogenetic Moran's I correlogram for description year in New World snakes. The analysis was done at five phylogenetic distance classes including equal numbers of species. (B) Description years (bars) structured into the phylogenetic tree. Colors represent the categories of threat used in the study; red: Threatened; green: Non-Threatened; blue: Data Deficient; and gray: Not-Evaluated. (TIF) [file pone.0113429.s001.tif]

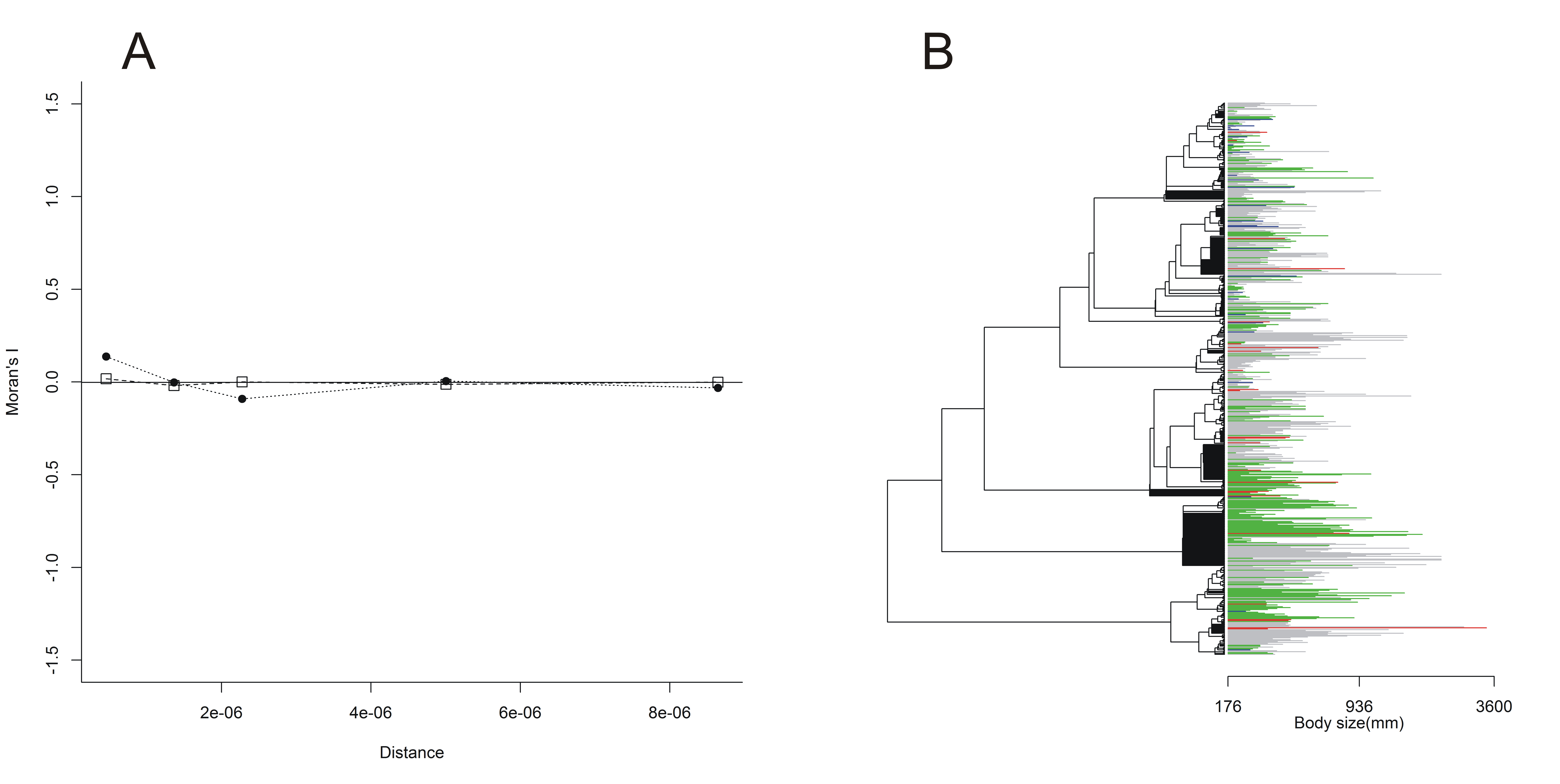

Supplement: Figure S2 — (A) Phylogenetic Moran's I correlogram for observed body sizes (dots) and residuals after phylogenetic control (squares) for New World snakes. The analysis was done at five phylogenetic distance classes including equal numbers of species. (B) Body size (bars) structured into the phylogenetic tree. Colors represent the categories of threat used in the study; red: Threatened; green: Non-Threatened; blue: Data Deficient; and gray: Not-Evaluated. (TIF) [file pone.0113429.s002.tif]
