# Supplementary material for: Body Size, Extinction Risk and Knowledge Bias in New World Snakes
Source: PLoS One. 2014 Nov 19;9(11):e113429. doi: 10.1371/journal.pone.0113429 (PMC4237443; doi:10.1371/journal.pone.0113429)
Supplement: Table S1 — Species IUCN red list conservation status (Following broader risk categories: TE = Threatened; NT = Non-Threatened; DD = Data Deficient; NE = Not-Evaluated) in 2012, maximum total length in millimeters and data source. (DOCX) [file pone.0113429.s003.docx]

**Table S1.** Species IUCN red list conservation status (Following broader risk categories: TE=Threatened; NT=Non-Threatened; DD=Data Deficient; NE=Not-Evaluated) in 2012, maximum total length in millimeters and data source.

| **Species** | **Conservation Status (2012)** | **Maximun total length (mm)** | **Source** |
| --- | --- | --- | --- |
| *Adelophis copei* | TE | 330 | Cope 1879 |
| *Adelophis foxi* | DD | 479 | Rossman & Wallach 1987 |
| *Adelphicos daryi* | TE | 574 | Kohler 2003 |
| *Adelphicos ibarrorum* | NE | 521 | Kohler 2003 |
| *Adelphicos latifasciatus* | NE | 437 | Kohler 2003 |
| *Adelphicos nigrilatum* | NT | 451 | Kohler 2003 |
| *Adelphicos quadrivirgatus* | NE | 390 | Kohler 2003 |
| *Adelphicos veraepacis* | NE | 461 | Kohler 2003 |
| *Agkistrodon bilineatus* | NT | 1380 | Terribile et al. 2009 |
| *Agkistrodon contortrix* | NT | 1346 | Terribile et al. 2009 |
| *Agkistrodon piscivorus* | NT | 1846 | Terribile et al. 2009 |
| *Agkistrodon taylori* | NT | 960 | Terribile et al. 2009 |
| *Alsophis antiguae* | TE | 990 | Daltry et al. 2001 |
| *Alsophis rijgersmaei* | TE | 993 | Cope 1879 |
| *Amastridium veliferum* | NE | 725 | Kohler 2003 |
| *Apostolepis arenaria* | NE | 300 | Freitas & Silva 2007 |
| *Apostolepis cearensis* | NE | 600 | Freitas & Silva 2005 |
| *Apostolepis gaboi* | NE | 300 | Freitas & Silva 2007 |
| *Apostolepis longicaudata* | NE | 500 | Freitas & Silva 2005 |
| *Apostolepis polylepis* | DD | 500 | Freitas & Silva 2007 |
| *Arizona elegans* | NT | 1780 | Boback & Guyer 2003 |
| *Atractus badius* | NE | 500 | Boback & Guyer 2003 |
| *Atractus clarki* | NE | 313 | Kohler 2003 |
| *Atractus darienensis* | NE | 346 | Kohler 2003 |
| *Atractus depressiocellus* | NE | 750 | Kohler 2003 |
| *Atractus duidensis* | NT | 500 | Boback & Guyer 2003 |
| *Atractus elaps* | NE | 500 | Boback & Guyer 2003 |
| *Atractus emigdioi* | NE | 415 | Boback & Guyer 2003 |
| *Atractus erythromelas* | NE | 600 | Boback & Guyer 2003 |
| *Atractus fuliginosus* | NE | 400 | Boback & Guyer 2003 |
| *Atractus guentheri* | NE | 300 | Freitas & Silva 2005 |
| *Atractus hostilitractus* | NE | 374 | Kohler 2003 |
| *Atractus insipidus* | NE | 235 | Boback & Guyer 2003 |
| *Atractus major* | NT | 723 | Boback & Guyer 2003 |
| *Atractus mariselae* | NE | 400 | Boback & Guyer 2003 |
| *Atractus modestus* | TE | 369 | Passo et al. 2009 |
| *Atractus nasutus* | NE | 199 | Passos et al. 2009 |
| *Atractus paisa* | NE | 401 | Passos et al. 2009 |
| *Atractus potschi* | NE | 300 | Freitas & Silva 2005 |
| *Atractus riveroi* | NE | 500 | Boback & Guyer 2003 |
| *Atractus steyermarki* | NE | 450 | Boback & Guyer 2003 |
| *Atractus titanicus* | NE | 473 | Passos et al. 2009 |
| *Atractus trilineatus* | NE | 350 | Boback & Guyer 2003 |
| *Atractus univittatus* | NE | 400 | Boback & Guyer 2003 |
| *Atractus ventrimaculatus* | NE | 500 | Boback & Guyer 2003 |
| *Atractus vittatus* | NE | 600 | Boback & Guyer 2003 |
| *Atropoides indomitus* | NE | 537 | Terribile et al. 2009 |
| *Atropoides mexicanus* | NE | 979 | Terribile et al. 2009 |
| *Atropoides nummifer* | NT | 695 | Terribile et al. 2009 |
| *Atropoides occiduus* | NE | 795 | Terribile et al. 2009 |
| *Atropoides olmec* | NT | 770 | Terribile et al. 2009 |
| *Atropoides picadoi* | NE | 1202 | Terribile et al. 2009 |
| *Bogertophis subocularis* | NT | 1680 | Boback & Guyer 2003 |
| *Boiruna maculata* | NE | 2000 | Boback & Guyer 2003 |
| *Boiruna sertaneja* | NE | 2000 | Freitas & Silva 2005 |
| *Bothriechis aurifer* | TE | 1010 | Terribile et al. 2009 |
| *Bothriechis bicolor* | NT | 1000 | Terribile et al. 2009 |
| *Bothriechis lateralis* | NE | 1000 | Terribile et al. 2009 |
| *Bothriechis marchi* | NE | 968 | Terribile et al. 2009 |
| *Bothriechis nigroviridis* | NE | 937 | Terribile et al. 2009 |
| *Bothriechis rowleyi* | TE | 973 | Terribile et al. 2009 |
| *Bothriechis schlegelii* | NE | 979 | Terribile et al. 2009 |
| *Bothriechis supraciliaris* | NE | 800 | Terribile et al. 2009 |
| *Bothriechis thalassinus* | NE | 967 | Terribile et al. 2009 |
| *Bothriopsis bilineata* | NE | 1230 | Terribile et al. 2009 |
| *Bothriopsis chloromelas* | NE | 1000 | Terribile et al. 2009 |
| *Bothriopsis medusa* | NE | 800 | Terribile et al. 2009 |
| *Bothriopsis oligolepis* | NT | 986 | Terribile et al. 2009 |
| *Bothriopsis pulchra* | NE | 764 | Terribile et al. 2009 |
| *Bothriopsis taeniata* | NE | 1750 | Terribile et al. 2009 |
| *Bothrocophias campbelli* | NE | 1230 | Terribile et al. 2009 |
| *Bothrocophias colombianus* | NE | 1360 | Terribile et al. 2009 |
| *Bothrocophias hyoprora* | NE | 830 | Terribile et al. 2009 |
| *Bothrocophias microphthalmus* | NE | 1162 | Terribile et al. 2009 |
| *Bothrocophias myersi* | NT | 756 | Terribile et al. 2009 |
| *Bothropoides alcatraz* | TE | 560 | Marques et al. 2002 |
| *Bothropoides diporus* | NE | 1100 | Terribile et al. 2009 |
| *Bothropoides erythromelas* | NT | 850 | Terribile et al. 2009 |
| *Bothropoides insularis* | TE | 947 | Marques et al. 2002 |
| *Bothropoides jararaca* | NE | 1600 | Terribile et al. 2009 |
| *Bothropoides lutzi* | NT | 800 | Terribile et al. 2009 |
| *Bothropoides matogrossensis* | NE | 1300 | Terribile et al. 2009 |
| *Bothropoides neuwiedi* | NE | 1000 | Terribile et al. 2009 |
| *Bothropoides pauloensis* | NE | 938 | Terribile et al. 2009 |
| *Bothropoides pubescens* | NE | 1200 | Terribile et al. 2009 |
| *Bothrops andianus* | NE | 1258 | Terribile et al. 2009 |
| *Bothrops asper* | NE | 2500 | Terribile et al. 2009 |
| *Bothrops atrox* | NE | 1541 | Terribile et al. 2009 |
| *Bothrops barnetti* | NE | 1400 | Terribile et al. 2009 |
| *Bothrops brazili* | NE | 1493 | Terribile et al. 2009 |
| *Bothrops jararacussu* | NE | 2200 | Terribile et al. 2009 |
| *Bothrops leucurus* | NE | 1200 | Terribile et al. 2009 |
| *Bothrops lojanus* | TE | 610 | Terribile et al. 2009 |
| *Bothrops marajoensis* | NE | 1500 | Terribile et al. 2009 |
| *Bothrops marmoratus* | NE | 800 | Terribile et al. 2009 |
| *Bothrops moojeni* | NE | 2300 | Terribile et al. 2009 |
| *Bothrops muriciensis* | NE | 884 | Terribile et al. 2009 |
| *Bothrops osbornei* | NE | 1400 | Terribile et al. 2009 |
| *Bothrops pictus* | NE | 1000 | Terribile et al. 2009 |
| *Bothrops pirajai* | TE | 1370 | Terribile et al. 2009 |
| *Bothrops punctatus* | NE | 1500 | Terribile et al. 2009 |
| *Bothrops sanctaecrucis* | NE | 665 | Terribile et al. 2009 |
| *Bothrops venezuelensis* | NE | 1667 | Terribile et al. 2009 |
| *Caaeteboia amarali* | NE | 400 | Freitas & Silva 2005 |
| *Calamodontophis ronaldoi* | TE | 345 | Franco et al. 2006 |
| *Carphophis amoenus* | NT | 340 | Boback & Guyer 2003 |
| *Carphophis vermis* | NT | 390 | Boback & Guyer 2003 |
| *Cemophora coccinea* | NT | 830 | Boback & Guyer 2003 |
| *Cerrophidion godmani* | NE | 822 | Terribile et al. 2009 |
| *Cerrophidion petlalcalensis* | DD | 467 | Terribile et al. 2009 |
| *Cerrophidion tzotzilorum* | NT | 500 | Terribile et al. 2009 |
| *Chapinophis xanthocheilus* | DD | 590 | Kohler 2003 |
| *Chilomeniscus stramineus* | NT | 250 | Boback & Guyer 2003 |
| *Chionactis occipitalis* | NT | 420 | Boback & Guyer 2003 |
| *Chionactis palarostris* | NT | 400 | Boback & Guyer 2003 |
| *Chironius bicarinatus* | NE | 1400 | Freitas & Silva 2005 |
| *Chironius carinatus* | NE | 2800 | Boback & Guyer 2003 |
| *Chironius exoletus* | NE | 1545 | Boback & Guyer 2003 |
| *Chironius flavolineatus* | NE | 1200 | Freitas & Silva 2005 |
| *Chironius fuscus* | NE | 2000 | Boback & Guyer 2003 |
| *Chironius grandisquamis* | NE | 2720 | Kohler 2003 |
| *Chironius laevicollis* | NE | 1800 | Freitas & Silva 2005 |
| *Chironius monticola* | NE | 1500 | Boback & Guyer 2003 |
| *Chironius multiventris* | NE | 2574 | Boback & Guyer 2003 |
| *Chironius quadricarinatus* | NE | 1000 | Freitas & Silva 2005 |
| *Chironius scurrulus* | NE | 2332 | Boback & Guyer 2003 |
| *Clelia clelia* | NE | 2600 | Boback & Guyer 2003 |
| *Clelia equatoriana* | NE | 1575 | Kohler 2003 |
| *Clelia plumbea* | NE | 2500 | Freitas & Silva 2005 |
| *Clelia rustica* | NE | 1200 | Boback & Guyer 2003 |
| *Clelia scytalina* | NE | 1800 | Boback & Guyer 2003 |
| *Clonophis kirtlandii* | NT | 620 | Boback & Guyer 2003 |
| *Coluber bilineatus* | NE | 1700 | Boback & Guyer 2003 |
| *Coluber constrictor* | NT | 1820 | Boback & Guyer 2003 |
| *Coluber flagellum* | NE | 2550 | Boback & Guyer 2003 |
| *Coluber lateralis* | NE | 1520 | Boback & Guyer 2003 |
| *Coluber mentovarius* | NE | 2546.1 | Boback & Guyer 2003 |
| *Coluber mormon* | NE | 1300 | Boback & Guyer 2003 |
| *Coluber schotti* | NE | 1680 | Boback & Guyer 2003 |
| *Coluber taeniatus* | NE | 1830 | Boback & Guyer 2003 |
| *Coniophanes alvarezi* | DD | 521 | Kohler 2003 |
| *Coniophanes bipunctatus* | NE | 770 | Boback & Guyer 2003 |
| *Coniophanes fissidens* | NE | 800 | Boback & Guyer 2003 |
| *Coniophanes imperialis* | NT | 550 | Kohler 2003 |
| *Coniophanes joanae* | NE | 369 | Kohler 2003 |
| *Coniophanes meridanus* | NT | 480 | Boback & Guyer 2003 |
| *Coniophanes piceivittis* | NT | 571 | Boback & Guyer 2003 |
| *Coniophanes quinquevittatus* | NT | 675 | Boback & Guyer 2003 |
| *Coniophanes schmidti* | NT | 680 | Boback & Guyer 2003 |
| *Conophis lineatus* | NT | 1170 | Boback & Guyer 2003 |
| *Conophis vittatus* | NT | 840 | Kohler 2003 |
| *Contia tenuis* | NT | 450 | Boback & Guyer 2003 |
| *Crisantophis nevermanni* | NE | 830 | Kohler 2003 |
| *Crotalus adamanteus* | NT | 2515 | Terribile et al. 2009 |
| *Crotalus aquilus* | NT | 678 | Terribile et al. 2009 |
| *Crotalus atrox* | NT | 2340 | Terribile et al. 2009 |
| *Crotalus basiliscus* | NT | 2045 | Terribile et al. 2009 |
| *Crotalus cerastes* | NT | 824 | Terribile et al. 2009 |
| *Crotalus durissus* | NT | 1800 | Terribile et al. 2009 |
| *Crotalus enyo* | NT | 898 | Terribile et al. 2009 |
| *Crotalus ericsmithi* | NE | 540 | Terribile et al. 2009 |
| *Crotalus horridus* | NT | 1892 | Terribile et al. 2009 |
| *Crotalus intermedius* | NT | 570 | Terribile et al. 2009 |
| *Crotalus lannomi* | DD | 638 | Terribile et al. 2009 |
| *Crotalus lepidus* | NT | 828 | Terribile et al. 2009 |
| *Crotalus mitchellii* | NT | 1367 | Terribile et al. 2009 |
| *Crotalus molossus* | NT | 1330 | Terribile et al. 2009 |
| *Crotalus oreganus* | NT | 1626 | Terribile et al. 2009 |
| *Crotalus polystictus* | NT | 1000 | Terribile et al. 2009 |
| *Crotalus pricei* | NT | 660 | Terribile et al. 2009 |
| *Crotalus pusillus* | TE | 682 | Terribile et al. 2009 |
| *Crotalus ravus* | NT | 700 | Terribile et al. 2009 |
| *Crotalus ruber* | NT | 1620 | Terribile et al. 2009 |
| *Crotalus scutulatus* | NT | 1373 | Terribile et al. 2009 |
| *Crotalus simus* | NE | 1800 | Terribile et al. 2009 |
| *Crotalus stejnegeri* | TE | 724 | Terribile et al. 2009 |
| *Crotalus tancitarensis* | DD | 410 | Terribile et al. 2009 |
| *Crotalus tigris* | NT | 885 | Terribile et al. 2009 |
| *Crotalus totonacus* | NE | 1665 | Terribile et al. 2009 |
| *Crotalus transversus* | NT | 465 | Terribile et al. 2009 |
| *Crotalus triseriatus* | NT | 683 | Terribile et al. 2009 |
| *Crotalus viridis* | NT | 1515 | Terribile et al. 2009 |
| *Crotalus willardi* | NT | 670 | Terribile et al. 2009 |
| *Dendrophidion dendrophis* | NE | 1070 | Boback & Guyer 2003 |
| *Dendrophidion nuchale* | NE | 1530 | Boback & Guyer 2003 |
| *Dendrophidion paucicarinatum* | NE | 1400 | Boback & Guyer 2003 |
| *Dendrophidion percarinatum* | NE | 1500 | Boback & Guyer 2003 |
| *Dendrophidion vinitor* | NT | 996 | Boback & Guyer 2003 |
| *Diadophis punctatus* | NT | 750 | Boback & Guyer 2003 |
| *Diaphorolepis wagneri* | NE | 600 | Kohler 2003 |
| *Dipsas albifrons* | TE | 600 | Freitas & Silva 2005 |
| *Dipsas articulata* | NE | 712 | Kohler 2003 |
| *Dipsas bicolor* | NE | 712 | Boback & Guyer 2003 |
| *Dipsas brevifacies* | NT | 590.8 | Boback & Guyer 2003 |
| *Dipsas catesbyi* | NT | 726 | Boback & Guyer 2003 |
| *Dipsas copei* | NE | 1000 | Boback & Guyer 2003 |
| *Dipsas indica* | NE | 1030 | Boback & Guyer 2003 |
| *Dipsas maxillaris* | DD | 405 | Kohler 2003 |
| *Dipsas pavonina* | NT | 1000 | Boback & Guyer 2003 |
| *Dipsas peruana* | NT | 1000 | Boback & Guyer 2003 |
| *Dipsas temporalis* | NE | 680 | Kohler 2003 |
| *Dipsas tenuissima* | NE | 555 | Boback & Guyer 2003 |
| *Dipsas variegata* | NE | 821 | Kohler 2003 |
| *Dipsas viguieri* | NT | 637 | Kohler 2003 |
| *Drepanoides anomalus* | NE | 600 | Boback & Guyer 2003 |
| *Drymarchon corais* | NE | 3000 | Boback & Guyer 2003 |
| *Drymobius chloroticus* | NT | 1200 | Kohler 2003 |
| *Drymobius margaritiferus* | NE | 1340 | Kohler 2003 |
| *Drymobius melanotropis* | NT | 1300 | Boback & Guyer 2003 |
| *Drymobius rhombifer* | NT | 1270 | Kohler 2003 |
| *Drymoluber brazili* | NE | 1000 | Freitas & Silva 2007 |
| *Drymoluber dichrous* | NE | 1500 | Boback & Guyer 2003 |
| *Elapomorphus lepidus* | NE | 600 | Freitas & Silva 2005 |
| *Elapomorphus spegazzinii* | NE | 370 | Boback & Guyer 2003 |
| *Elapomorphus wuchereri* | NE | 1400 | Freitas & Silva 2005 |
| *Enuliophis sclateri* | NE | 550 | Boback & Guyer 2003 |
| *Enulius bifoveatus* | NE | 321 | Kohler 2003 |
| *Enulius flavitorques* | NE | 500 | Boback & Guyer 2003 |
| *Enulius roatanensis* | NE | 346 | Kohler 2003 |
| *Erythrolamprus aesculapii* | NE | 1500 | Boback & Guyer 2003 |
| *Erythrolamprus bizonus* | NT | 1500 | Boback & Guyer 2003 |
| *Erythrolamprus mimus* | NE | 1000 | Kohler 2003 |
| *Erythrolamprus ocellatus* | NE | 545 | Boback & Guyer 2003 |
| *Erythrolamprus pseudocorallus* | NE | 1000 | Boback & Guyer 2003 |
| *Erythrolamprus pygmaea* | NE | 250 | Boback & Guyer 2003 |
| *Farancia abacura* | NT | 2070 | Boback & Guyer 2003 |
| *Farancia erytrogramma* | NT | 1680 | Boback & Guyer 2003 |
| *Ficimia publia* | NE | 461 | Kohler 2003 |
| *Ficimia ramirezi* | DD | 313 | Kohler 2003 |
| *Ficimia streckeri* | NT | 480 | Boback & Guyer 2003 |
| *Geophis bellus* | NE | 201 | Kohler 2003 |
| *Geophis brachycephalus* | NT | 460 | Boback & Guyer 2003 |
| *Geophis cancellatus* | NT | 410 | Kohler 2003 |
| *Geophis carinosus* | NT | 276 | Kohler 2003 |
| *Geophis championi* | NE | 255 | Kohler 2003 |
| *Geophis damiani* | NE | 327 | Kohler 2003 |
| *Geophis downsi* | NE | 248 | Boback & Guyer 2003 |
| *Geophis dunni* | DD | 367 | Kohler 2003 |
| *Geophis fulvoguttatus* | NE | 398 | Kohler 2003 |
| *Geophis godmani* | NE | 401 | Boback & Guyer 2003 |
| *Geophis hoffmanni* | NE | 300 | Boback & Guyer 2003 |
| *Geophis immaculatus* | NT | 305 | Kohler 2003 |
| *Geophis laticinctus* | NT | 384 | Kohler 2003 |
| *Geophis nasalis* | NT | 350 | Kohler 2003 |
| *Geophis rhodogaster* | NT | 377 | Kohler 2003 |
| *Geophis ruthveni* | NT | 260 | Boback & Guyer 2003 |
| *Geophis talamancae* | NE | 218 | Boback & Guyer 2003 |
| *Geophis zeledoni* | NE | 417 | Boback & Guyer 2003 |
| *Gyalopion canum* | NT | 360 | Boback & Guyer 2003 |
| *Gyalopion quadrangulare* | NT | 300 | Boback & Guyer 2003 |
| *Helicops angulatus* | NE | 1000 | Boback & Guyer 2003 |
| *Helicops leopardinus* | NE | 1150 | Freitas & Silva 2005 |
| *Helicops petersi* | NE | 669 | Boback & Guyer 2003 |
| *Helicops polylepis* | NE | 1040 | Boback & Guyer 2003 |
| *Helicops scalaris* | NT | 1000 | Boback & Guyer 2003 |
| *Helicops yacu* | NE | 750 | Boback & Guyer 2003 |
| *Heterodon nasicus* | NT | 1010 | Boback & Guyer 2003 |
| *Heterodon platirhinos* | NT | 1160 | Boback & Guyer 2003 |
| *Heterodon simus* | TE | 610 | Boback & Guyer 2003 |
| *Hydromorphus concolor* | NE | 797 | Boback & Guyer 2003 |
| *Hydromorphus dunni* | NE | 482 | Kohler 2003 |
| *Hydrops martii* | NT | 1160 | Boback & Guyer 2003 |
| *Hydrops triangularis* | NE | 850 | Boback & Guyer 2003 |
| *Hypsiglena torquata* | NT | 650 | Boback & Guyer 2003 |
| *Imantodes cenchoa* | NE | 1500 | Boback & Guyer 2003 |
| *Imantodes gemmistratus* | NE | 880 | Boback & Guyer 2003 |
| *Imantodes inornatus* | NT | 1035 | Boback & Guyer 2003 |
| *Imantodes lentiferus* | NE | 1040 | Boback & Guyer 2003 |
| *Imantodes phantasma* | DD | 1082 | Kohler 2003 |
| *Imantodes tenuissimus* | NE | 870 | Boback & Guyer 2003 |
| *Lachesis acrochorda* | NE | 3000 | Terribile et al. 2009 |
| *Lachesis melanocephala* | NE | 2400 | Terribile et al. 2009 |
| *Lachesis muta* | TE | 3600 | Terribile et al. 2009 |
| *Lachesis stenophrys* | NE | 3300 | Terribile et al. 2009 |
| *Lampropeltis alterna* | NT | 1470 | Boback & Guyer 2003 |
| *Lampropeltis calligaster* | NT | 1430 | Boback & Guyer 2003 |
| *Lampropeltis extenuatum* | NE | 650 | Boback & Guyer 2003 |
| *Lampropeltis getula* | NT | 2080 | Boback & Guyer 2003 |
| *Lampropeltis pyromelana* | NT | 1040 | Boback & Guyer 2003 |
| *Lampropeltis ruthveni* | NT | 630 | Roth-Monzón et al. 2011 |
| *Lampropeltis triangulum* | NE | 2000 | Kohler 2003 |
| *Lampropeltis zonata* | NT | 1020 | Boback & Guyer 2003 |
| *Leptodeira annulata* | NE | 900 | Freitas & Silva 2005 |
| *Leptodeira frenata* | NT | 715 | Kohler 2003 |
| *Leptodeira nigrofasciata* | NT | 581 | Boback & Guyer 2003 |
| *Leptodeira rubricata* | NE | 700 | Boback & Guyer 2003 |
| *Leptodeira septentrionalis* | NE | 1055 | Kohler 2003 |
| *Leptodrymus pulcherrimus* | NE | 1600 | Boback & Guyer 2003 |
| *Leptophis ahaetulla* | NE | 2250 | Boback & Guyer 2003 |
| *Leptophis depressirostris* | NE | 1500 | Boback & Guyer 2003 |
| *Leptophis diplotropis* | NT | 1410 | Kohler 2003 |
| *Leptophis mexicanus* | NE | 1380 | Boback & Guyer 2003 |
| *Leptophis modestus* | TE | 1720 | Kohler 2003 |
| *Leptophis nebulosus* | NE | 854 | Kohler 2003 |
| *Leptophis riveti* | NE | 1000 | Boback & Guyer 2003 |
| *Leptophis stimsoni* | NE | 300 | Boback & Guyer 2003 |
| *Liophis almadensis* | NE | 600 | Freitas & Silva 2005 |
| *Liophis breviceps* | NE | 900 | Boback & Guyer 2003 |
| *Liophis cobella* | NE | 795 | Boback & Guyer 2003 |
| *Liophis epinephelus* | NE | 800 | Boback & Guyer 2003 |
| *Liophis ingeri* | NE | 700 | Boback & Guyer 2003 |
| *Liophis maryellenae* | NE | 600 | Freitas & Silva 2007 |
| *Liophis melanotus* | NT | 700 | Boback & Guyer 2003 |
| *Liophis miliaris* | NE | 900 | Boback & Guyer 2003 |
| *Liophis mossoroensis* | NE | 610 | Boback & Guyer 2003 |
| *Liophis poecilogyrus* | NE | 900 | Freitas & Silva 2005 |
| *Liophis reginae* | NE | 800 | Boback & Guyer 2003 |
| *Liophis sagittifer* | NE | 1020 | Boback & Guyer 2003 |
| *Liophis taeniogaster* | NE | 700 | Freitas & Silva 2005 |
| *Liophis trebbaui* | NE | 600 | Boback & Guyer 2003 |
| *Liophis typhlus* | NE | 700 | Boback & Guyer 2003 |
| *Liophis viridis* | NT | 700 | Freitas & Silva 2005 |
| *Lygophis anomalus* | NE | 700 | Boback & Guyer 2003 |
| *Lygophis dilepis* | NT | 800 | Freitas & Silva 2005 |
| *Lygophis lineatus* | NE | 750 | Boback & Guyer 2003 |
| *Manolepis putnami* | NT | 717 | Kohler 2003 |
| *Mastigodryas amarali* | NE | 1500 | Boback & Guyer 2003 |
| *Mastigodryas bifossatus* | NE | 2000 | Boback & Guyer 2003 |
| *Mastigodryas boddaerti* | NE | 1600 | Boback & Guyer 2003 |
| *Mastigodryas dorsalis* | NE | 1140 | Kohler 2003 |
| *Mastigodryas melanolomus* | NT | 1515 | Boback & Guyer 2003 |
| *Mastigodryas pleei* | NE | 1500 | Boback & Guyer 2003 |
| *Micruroides euryxanthus* | NT | 615 | Terribile et al. 2009 |
| *Micrurus albicinctus* | NE | 573 | Terribile et al. 2009 |
| *Micrurus alleni* | NE | 1340 | Terribile et al. 2009 |
| *Micrurus altirostris* | NE | 1310 | Terribile et al. 2009 |
| *Micrurus ancoralis* | NE | 1486 | Terribile et al. 2009 |
| *Micrurus annellatus* | NE | 728 | Terribile et al. 2009 |
| *Micrurus averyi* | NE | 715 | Terribile et al. 2009 |
| *Micrurus baliocoryphus* | NE | 1449 | Terribile et al. 2009 |
| *Micrurus bernadi* | NT | 826 | Terribile et al. 2009 |
| *Micrurus bocourti* | NE | 820 | Terribile et al. 2009 |
| *Micrurus bogerti* | DD | 770 | Terribile et al. 2009 |
| *Micrurus brasiliensis* | NE | 1513 | Terribile et al. 2009 |
| *Micrurus browni* | NT | 1000 | Terribile et al. 2009 |
| *Micrurus camilae* | NE | 291.8 | Terribile et al. 2009 |
| *Micrurus catamayensis* | NE | 915 | Terribile et al. 2009 |
| *Micrurus circinalis* | NE | 537 | Terribile et al. 2009 |
| *Micrurus clarki* | NE | 832 | Terribile et al. 2009 |
| *Micrurus collaris* | NE | 456 | Terribile et al. 2009 |
| *Micrurus corallinus* | NE | 987 | Terribile et al. 2009 |
| *Micrurus decoratus* | NE | 670 | Terribile et al. 2009 |
| *Micrurus diana* | NE | 1008 | Terribile et al. 2009 |
| *Micrurus diastema* | NT | 900 | Terribile et al. 2009 |
| *Micrurus dissoleucus* | NT | 650 | Terribile et al. 2009 |
| *Micrurus distans* | NT | 1075 | Terribile et al. 2009 |
| *Micrurus dumerilii* | NE | 948 | Terribile et al. 2009 |
| *Micrurus elegans* | NT | 1000 | Terribile et al. 2009 |
| *Micrurus ephippifer* | TE | 926 | Terribile et al. 2009 |
| *Micrurus filiformis* | NE | 960 | Terribile et al. 2009 |
| *Micrurus frontalis* | NE | 1418 | Terribile et al. 2009 |
| *Micrurus fulvius* | NT | 1295 | Terribile et al. 2009 |
| *Micrurus hemprichii* | NE | 917 | Terribile et al. 2009 |
| *Micrurus hippocrepis* | NE | 710 | Terribile et al. 2009 |
| *Micrurus ibiboboca* | NE | 1330 | Terribile et al. 2009 |
| *Micrurus isozonus* | NT | 1500 | Terribile et al. 2009 |
| *Micrurus langsdorffi* | NT | 770 | Terribile et al. 2009 |
| *Micrurus laticollaris* | NT | 800 | Terribile et al. 2009 |
| *Micrurus latifasciatus* | NT | 1140 | Terribile et al. 2009 |
| *Micrurus lemniscatus* | NE | 1450 | Terribile et al. 2009 |
| *Micrurus limbatus* | NT | 735 | Terribile et al. 2009 |
| *Micrurus margaritiferus* | NE | 773 | Terribile et al. 2009 |
| *Micrurus medemi* | NE | 666 | Terribile et al. 2009 |
| *Micrurus meridensis* | NE | 390 | Terribile et al. 2009 |
| *Micrurus mertensi* | NE | 1115 | Terribile et al. 2009 |
| *Micrurus mipartitus* | NE | 1200 | Terribile et al. 2009 |
| *Micrurus multifasciatus* | NE | 1200 | Terribile et al. 2009 |
| *Micrurus multiscutatus* | DD | 842 | Terribile et al. 2009 |
| *Micrurus narduccii* | NE | 1157 | Terribile et al. 2009 |
| *Micrurus nebularis* | DD | 557 | Terribile et al. 2009 |
| *Micrurus nigrocinctus* | NE | 1150 | Terribile et al. 2009 |
| *Micrurus oligoanellatus* | NE | 625 | Terribile et al. 2009 |
| *Micrurus ornatissimus* | NE | 848 | Terribile et al. 2009 |
| *Micrurus pacaraimae* | NE | 355 | Terribile et al. 2009 |
| *Micrurus pachecogili* | DD | 639 | Terribile et al. 2009 |
| *Micrurus paraensis* | NT | 530 | Terribile et al. 2009 |
| *Micrurus peruvianus* | NE | 543 | Terribile et al. 2009 |
| *Micrurus petersi* | NE | 667 | Terribile et al. 2009 |
| *Micrurus proximans* | NT | 565 | Terribile et al. 2009 |
| *Micrurus psyches* | NE | 910 | Terribile et al. 2009 |
| *Micrurus putumayensis* | NE | 805 | Terribile et al. 2009 |
| *Micrurus pyrrhocryptus* | NT | 1241 | Terribile et al. 2009 |
| *Micrurus remotus* | NE | 567 | Terribile et al. 2009 |
| *Micrurus renjifoi* | NE | 427 | Terribile et al. 2009 |
| *Micrurus sangilensis* | NE | 600 | Terribile et al. 2009 |
| *Micrurus scutiventris* | NE | 445 | Terribile et al. 2009 |
| *Micrurus serranus* | NE | 822 | Terribile et al. 2009 |
| *Micrurus silviae* | NE | 1333 | Terribile et al. 2009 |
| *Micrurus spixii* | NE | 1400 | Terribile et al. 2009 |
| *Micrurus spurrelli* | NE | 633 | Terribile et al. 2009 |
| *Micrurus steindachneri* | NE | 880 | Terribile et al. 2009 |
| *Micrurus stewarti* | NE | 833 | Terribile et al. 2009 |
| *Micrurus stuarti* | NE | 745 | Terribile et al. 2009 |
| *Micrurus surinamensis* | NE | 1350 | Terribile et al. 2009 |
| *Micrurus tamaulipensis* | DD | 678 | Terribile et al. 2009 |
| *Micrurus tener* | NT | 1217 | Terribile et al. 2009 |
| *Micrurus tricolor* | NE | 1181 | Terribile et al. 2009 |
| *Micrurus tschudii* | NE | 880 | Terribile et al. 2009 |
| *Mixcoatlus barbouri* | TE | 510 | Terribile et al. 2009 |
| *Mixcoatlus melanurus* | TE | 578 | Terribile et al. 2009 |
| *Mussurana bicolor* | NT | 1000 | Boback & Guyer 2003 |
| *Nerodia clarkii* | NT | 930 | Boback & Guyer 2003 |
| *Nerodia cyclopion* | NT | 1270 | Boback & Guyer 2003 |
| *Nerodia erythrogaster* | NT | 1570 | Boback & Guyer 2003 |
| *Nerodia fasciata* | NT | 1590 | Boback & Guyer 2003 |
| *Nerodia floridana* | NT | 1880 | Boback & Guyer 2003 |
| *Nerodia harteri* | NT | 900 | Boback & Guyer 2003 |
| *Nerodia paucimaculata* | NT | 900 | Boback & Guyer 2003 |
| *Nerodia rhombifer* | NT | 1600 | Boback & Guyer 2003 |
| *Nerodia sipedon* | NT | 1500 | Boback & Guyer 2003 |
| *Nerodia taxispilota* | NT | 1770 | Boback & Guyer 2003 |
| *Ninia atrata* | NE | 500 | Boback & Guyer 2003 |
| *Ninia celata* | NE | 450 | Boback & Guyer 2003 |
| *Ninia diademata* | NT | 421 | Kohler 2003 |
| *Ninia espinali* | NT | 510 | Kohler 2003 |
| *Ninia hudsoni* | NE | 409 | Boback & Guyer 2003 |
| *Ninia maculata* | NE | 352 | Boback & Guyer 2003 |
| *Ninia pavimentata* | NE | 380 | Kohler 2003 |
| *Ninia psephota* | NE | 494 | Boback & Guyer 2003 |
| *Ninia sebae* | NE | 386 | Boback & Guyer 2003 |
| *Nothopsis rugosus* | NT | 433 | Boback & Guyer 2003 |
| *Omoadiphas aurula* | NE | 210 | Kohler 2003 |
| *Opheodrys aestivus* | NT | 1160 | Boback & Guyer 2003 |
| *Opheodrys vernalis* | NE | 650 | Boback & Guyer 2003 |
| *Ophryacus undulatus* | TE | 700 | Terribile et al. 2009 |
| *Oxybelis aeneus* | NE | 1900 | Boback & Guyer 2003 |
| *Oxybelis brevirostris* | NE | 1200 | Boback & Guyer 2003 |
| *Oxybelis fulgidus* | NE | 2400 | Boback & Guyer 2003 |
| *Oxybelis wilsoni* | NE | 1982 | Kohler 2003 |
| *Oxyrhopus clathratus* | NE | 800 | Freitas & Silva 2005 |
| *Oxyrhopus formosus* | NE | 1027 | Boback & Guyer 2003 |
| *Oxyrhopus guibei* | NE | 1200 | Freitas & Silva 2005 |
| *Oxyrhopus melanogenys* | NT | 1018 | Boback & Guyer 2003 |
| *Oxyrhopus petolarius* | NE | 2200 | Kohler 2003 |
| *Oxyrhopus rhombifer* | NE | 900 | Boback & Guyer 2003 |
| *Oxyrhopus trigeminus* | NE | 1110 | Boback & Guyer 2003 |
| *Pantherophis bairdi* | NT | 1570 | Boback & Guyer 2003 |
| *Pantherophis emoryi* | NT | 1530 | Boback & Guyer 2003 |
| *Pantherophis guttatus* | NT | 1830 | Boback & Guyer 2003 |
| *Pantherophis obsoletus* | NT | 2560 | Boback & Guyer 2003 |
| *Pantherophis vulpinus* | NT | 1790 | Boback & Guyer 2003 |
| *Phalotris cuyanus* | NE | 450 | Boback & Guyer 2003 |
| *Phalotris punctatus* | NE | 430 | Boback & Guyer 2003 |
| *Philodryas aestiva* | NE | 1000 | Freitas & Silva 2007 |
| *Philodryas baroni* | NE | 1000 | Boback & Guyer 2003 |
| *Philodryas chamissonis* | DD | 1050 | Boback & Guyer 2003 |
| *Philodryas nattereri* | NE | 1800 | Freitas & Silva 2005 |
| *Philodryas olfersii* | NE | 1400 | Freitas & Silva 2005 |
| *Philodryas patagoniensis* | NE | 1600 | Freitas & Silva 2005 |
| *Philodryas psammophidea* | NT | 1000 | Boback & Guyer 2003 |
| *Philodryas tachymenoides* | NT | 1060 | Boback & Guyer 2003 |
| *Philodryas viridissima* | NE | 1500 | Boback & Guyer 2003 |
| *Phimophis chui* | NE | 400 | Freitas & Silva 2007 |
| *Phimophis guerini* | NE | 750 | Freitas & Silva 2005 |
| *Phimophis guianensis* | NE | 960 | Kohler 2003 |
| *Phimophis iglesiasi* | NE | 500 | Freitas & Silva 2007 |
| *Phimophis scriptorcibatus* | NE | 300 | Freitas & Silva 2007 |
| *Phimophis vittatus* | NE | 700 | Boback & Guyer 2003 |
| *Phyllorhynchus browni* | NT | 510 | Boback & Guyer 2003 |
| *Phyllorhynchus decurtatus* | NT | 500 | Boback & Guyer 2003 |
| *Pituophis catenifer* | NT | 2750 | Boback & Guyer 2003 |
| *Pituophis lineaticollis* | NT | 2100 | Kohler 2003 |
| *Pituophis melanoleucus* | NT | 2540 | Boback & Guyer 2003 |
| *Pituophis ruthveni* | TE | 1780 | Boback & Guyer 2003 |
| *Plesiodipsas perijanensis* | DD | 910 | Boback & Guyer 2003 |
| *Pliocercus elapoides* | NT | 657 | Kohler 2003 |
| *Pliocercus euryzonus* | NT | 1300 | Kohler 2003 |
| *Porthidium arcosae* | NE | 635 | Terribile et al. 2009 |
| *Porthidium dunni* | NT | 570 | Terribile et al. 2009 |
| *Porthidium hespere* | DD | 579 | Terribile et al. 2009 |
| *Porthidium lansbergii* | NE | 900 | Terribile et al. 2009 |
| *Porthidium nasutum* | NT | 600 | Terribile et al. 2009 |
| *Porthidium ophryomegas* | NE | 770 | Terribile et al. 2009 |
| *Porthidium porrasi* | NE | 700 | Terribile et al. 2009 |
| *Porthidium volcanicum* | NE | 536 | Terribile et al. 2009 |
| *Porthidium yucatanicum* | NT | 598 | Terribile et al. 2009 |
| *Pseudalsophis elegans* | NT | 770 | Boback & Guyer 2003 |
| *Pseudelaphe flavirufa* | NT | 1650 | Kohler 2003 |
| *Pseudoboa coronata* | NE | 1080 | Boback & Guyer 2003 |
| *Pseudoboa neuwiedii* | NE | 1200 | Boback & Guyer 2003 |
| *Pseudoboa nigra* | NE | 1450 | Freitas & Silva 2005 |
| *Pseudoeryx plicatilis* | NT | 1440 | Boback & Guyer 2003 |
| *Pseudotomodon trigonatus* | NE | 450 | Boback & Guyer 2003 |
| *Pseustes poecilonotus* | NT | 2100 | Kohler 2003 |
| *Pseustes sulphureus* | NE | 3000 | Boback & Guyer 2003 |
| *Psomophis joberti* | NE | 400 | Freitas & Silva 2005 |
| *Regina alleni* | NT | 650 | Boback & Guyer 2003 |
| *Regina grahami* | NE | 1190 | Boback & Guyer 2003 |
| *Regina rigida* | NT | 800 | Boback & Guyer 2003 |
| *Regina septemvittata* | NT | 920 | Boback & Guyer 2003 |
| *Rhadinaea calligaster* | NE | 513 | Boback & Guyer 2003 |
| *Rhadinaea decorata* | NE | 470 | Boback & Guyer 2003 |
| *Rhadinaea flavilata* | NT | 400 | Boback & Guyer 2003 |
| *Rhadinaea macdougalli* | DD | 292 | Kohler 2003 |
| *Rhadinaea pulveriventris* | NE | 502 | Boback & Guyer 2003 |
| *Rhadinaea sargenti* | NE | 310 | Kohler 2003 |
| *Rhadinaea vermiculaticeps* | NE | 374 | Kohler 2003 |
| *Rhadinella anachoreta* | NE | 251 | Kohler 2003 |
| *Rhadinella godmani* | NE | 568 | Boback & Guyer 2003 |
| *Rhadinella hannsteini* | DD | 393 | Kohler 2003 |
| *Rhadinella hempsteadae* | NE | 593 | Kohler 2003 |
| *Rhadinella kanalchutchan* | DD | 490 | Kohler 2003 |
| *Rhadinella kinkelini* | NT | 376 | Kohler 2003 |
| *Rhadinella lachrymans* | NT | 493 | Kohler 2003 |
| *Rhadinella montecristi* | NE | 555 | Kohler 2003 |
| *Rhadinella pilonaorum* | NE | 310 | Kohler 2003 |
| *Rhadinella posadasi* | NE | 288 | Kohler 2003 |
| *Rhadinella rogerromani* | NE | 286 | Kohler 2003 |
| *Rhadinella serperaster* | DD | 445 | Boback & Guyer 2003 |
| *Rhadinella tolpanorum* | NE | 381 | Kohler 2003 |
| *Rhinobothryum bovallii* | NT | 1760 | Boback & Guyer 2003 |
| *Rhinocerophis alternatus* | NE | 1690 | Terribile et al. 2009 |
| *Rhinocerophis ammodytoides* | NE | 1000 | Terribile et al. 2009 |
| *Rhinocerophis cotiara* | NE | 945 | Terribile et al. 2009 |
| *Rhinocerophis fonsecai* | NE | 1079 | Terribile et al. 2009 |
| *Rhinocerophis itapetiningae* | NT | 500 | Terribile et al. 2009 |
| *Rhinocerophis jonathani* | NE | 881 | Terribile et al. 2009 |
| *Rhinocheilus lecontei* | NT | 1040 | Boback & Guyer 2003 |
| *Salvadora deserticola* | NE | 1020 | Boback & Guyer 2003 |
| *Salvadora grahamiae* | NT | 1190 | Boback & Guyer 2003 |
| *Salvadora hexalepis* | NT | 1150 | Boback & Guyer 2003 |
| *Salvadora lemniscata* | NT | 1300 | Kohler 2003 |
| *Scaphiodontophis annulatus* | NE | 920 | Boback & Guyer 2003 |
| *Scolecophis atrocinctus* | NE | 450 | Boback & Guyer 2003 |
| *Seminatrix pygaea* | NT | 470 | Boback & Guyer 2003 |
| *Senticolis triaspis* | NE | 1350 | Boback & Guyer 2003 |
| *Sibon annulatus* | NE | 557 | Boback & Guyer 2003 |
| *Sibon anthracops* | NE | 664 | Kohler 2003 |
| *Sibon argus* | NE | 690 | Boback & Guyer 2003 |
| *Sibon carri* | NE | 408 | Kohler 2003 |
| *Sibon dimidiatus* | NT | 750 | Boback & Guyer 2003 |
| *Sibon longifrenis* | NE | 624 | Boback & Guyer 2003 |
| *Sibon nebulata* | NE | 830 | Boback & Guyer 2003 |
| *Sibon sanniolus* | NT | 448 | Boback & Guyer 2003 |
| *Sibynomorphus mikanii* | NE | 400 | Freitas & Silva 2005 |
| *Sibynomorphus neuwiedi* | NE | 600 | Freitas & Silva 2005 |
| *Simophis rhinostoma* | NE | 600 | Freitas & Silva 2005 |
| *Siphlophis cervinus* | NE | 750 | Boback & Guyer 2003 |
| *Siphlophis compressus* | NT | 1200 | Freitas & Silva 2005 |
| *Siphlophis leucocephalus* | NT | 900 | Freitas & Silva 2005 |
| *Siphlophis pulcher* | NT | 1000 | Freitas & Silva 2005 |
| *Sistrurus catenatus* | NT | 925 | Terribile et al. 2009 |
| *Sistrurus miliarius* | NT | 788 | Terribile et al. 2009 |
| *Sonora semiannulata* | NT | 480 | Boback & Guyer 2003 |
| *Spilotes pullatus* | NE | 3000 | Freitas & Silva 2005 |
| *Stenorrhina degenhardtii* | NE | 870.55 | Boback & Guyer 2003 |
| *Stenorrhina freminvillei* | NT | 821.1 | Boback & Guyer 2003 |
| *Storeria dekayi* | NT | 540 | Boback & Guyer 2003 |
| *Storeria occipitomaculata* | NT | 410 | Boback & Guyer 2003 |
| *Symphimus leucostomus* | NT | 810 | Kohler 2003 |
| *Symphimus mayae* | NT | 890 | Kohler 2003 |
| *Tachymenis chilensis* | NT | 410 | Boback & Guyer 2003 |
| *Tachymenis peruviana* | NE | 460 | Boback & Guyer 2003 |
| *Taeniophallus affinis* | NT | 500 | Freitas & Silva 2005 |
| *Taeniophallus brevirostris* | NE | 391 | Boback & Guyer 2003 |
| *Taeniophallus occipitalis* | NE | 530 | Boback & Guyer 2003 |
| *Tantilla albiceps* | NE | 212 | Kohler 2003 |
| *Tantilla alticola* | NE | 327 | Boback & Guyer 2003 |
| *Tantilla armillata* | NE | 490 | Boback & Guyer 2003 |
| *Tantilla atriceps* | NT | 230 | Boback & Guyer 2003 |
| *Tantilla bairdi* | DD | 455 | Kohler 2003 |
| *Tantilla brevicauda* | NE | 1510 | Kohler 2003 |
| *Tantilla coronata* | NT | 330 | Boback & Guyer 2003 |
| *Tantilla cucullata* | NT | 650 | Boback & Guyer 2003 |
| *Tantilla cuniculator* | NT | 220 | Kohler 2003 |
| *Tantilla gracilis* | NT | 250 | Boback & Guyer 2003 |
| *Tantilla hobartsmithi* | NT | 370 | Boback & Guyer 2003 |
| *Tantilla impensa* | NT | 725 | Kohler 2003 |
| *Tantilla jani* | DD | 242 | Kohler 2003 |
| *Tantilla lempira* | NE | 254 | Kohler 2003 |
| *Tantilla marcovani* | NE | 400 | Freitas & Silva 2007 |
| *Tantilla melanocephala* | NE | 500 | Boback & Guyer 2003 |
| *Tantilla moesta* | NT | 612.5 | Boback & Guyer 2003 |
| *Tantilla nigriceps* | NT | 380 | Boback & Guyer 2003 |
| *Tantilla oolitica* | TE | 290 | Boback & Guyer 2003 |
| *Tantilla planiceps* | NT | 390 | Boback & Guyer 2003 |
| *Tantilla relicta* | NT | 230 | Boback & Guyer 2003 |
| *Tantilla reticulata* | NE | 312 | Boback & Guyer 2003 |
| *Tantilla robusta* | DD | 426 | Canseco-Marquez et al. 2002 |
| *Tantilla rubra* | NT | 594 | Kohler 2003 |
| *Tantilla ruficeps* | NE | 500 | Boback & Guyer 2003 |
| *Tantilla schistosa* | NE | 350 | Boback & Guyer 2003 |
| *Tantilla semicincta* | NE | 600 | Boback & Guyer 2003 |
| *Tantilla shawi* | TE | 690 | Campbell et al. 1995 |
| *Tantilla supracincta* | NE | 590 | Boback & Guyer 2003 |
| *Tantilla taeniata* | NE | 415 | Kohler 2003 |
| *Tantilla tayrae* | DD | 317 | Kohler 2003 |
| *Tantilla tecta* | NE | 222 | Kohler 2003 |
| *Tantilla triseriata* | DD | 203 | Kohler 2003 |
| *Tantilla tritaeniata* | NE | 273 | Kohler 2003 |
| *Tantilla vermiformis* | DD | 520 | Kohler 2003 |
| *Tantilla vulcani* | NE | 246 | Kohler 2003 |
| *Tantilla wilcoxi* | NT | 360 | Boback & Guyer 2003 |
| *Tantilla yaquia* | NT | 320 | Boback & Guyer 2003 |
| *Tantillita brevissima* | NT | 150 | Kohler 2003 |
| *Tantillita canula* | NT | 175 | Kohler 2003 |
| *Tantillita lintoni* | NT | 200 | Kohler 2003 |
| *Thamnodynastes almae* | NE | 400 | Freitas & Silva 2007 |
| *Thamnodynastes chimanta* | NE | 300 | Boback & Guyer 2003 |
| *Thamnodynastes hypoconia* | NE | 700 | Boback & Guyer 2003 |
| *Thamnodynastes pallidus* | NT | 600 | Boback & Guyer 2003 |
| *Thamnodynastes sertanejo* | NE | 600 | Freitas & Silva 2007 |
| *Thamnophis atratus* | NT | 1020 | Boback & Guyer 2003 |
| *Thamnophis brachystoma* | NT | 560 | Boback & Guyer 2003 |
| *Thamnophis butleri* | NT | 737 | Boback & Guyer 2003 |
| *Thamnophis chrysocephalus* | NT | 692 | Boback & Guyer 2003 |
| *Thamnophis couchii* | NT | 1600 | Boback & Guyer 2003 |
| *Thamnophis cyrtopsis* | NT | 1145 | Kohler 2003 |
| *Thamnophis elegans* | NT | 1070 | Boback & Guyer 2003 |
| *Thamnophis eques* | NT | 1120 | Boback & Guyer 2003 |
| *Thamnophis exsul* | NT | 463 | Boback & Guyer 2003 |
| *Thamnophis fulvus* | NT | 808 | Boback & Guyer 2003 |
| *Thamnophis gigas* | TE | 1630 | Boback & Guyer 2003 |
| *Thamnophis godmani* | NT | 669 | Boback & Guyer 2003 |
| *Thamnophis hammondii* | NT | 1050 | Boback & Guyer 2003 |
| *Thamnophis marcianus* | NE | 1090 | Kohler 2003 |
| *Thamnophis melanogaster* | TE | 864 | Boback & Guyer 2003 |
| *Thamnophis mendax* | TE | 710 | Boback & Guyer 2003 |
| *Thamnophis nigronuchalis* | DD | 767 | Boback & Guyer 2003 |
| *Thamnophis ordinoides* | NT | 965 | Boback & Guyer 2003 |
| *Thamnophis postremus* | NT | 747 | Boback & Guyer 2003 |
| *Thamnophis proximus* | NE | 1250 | Boback & Guyer 2003 |
| *Thamnophis pulchrilatus* | NT | 722 | Boback & Guyer 2003 |
| *Thamnophis radix* | NT | 1100 | Boback & Guyer 2003 |
| *Thamnophis rufipunctatus* | NT | 953 | Boback & Guyer 2003 |
| *Thamnophis sauritus* | NT | 1020 | Boback & Guyer 2003 |
| *Thamnophis scalaris* | NT | 801 | Boback & Guyer 2003 |
| *Thamnophis scaliger* | TE | 567 | Boback & Guyer 2003 |
| *Thamnophis sirtalis* | NT | 1372 | Boback & Guyer 2003 |
| *Thamnophis sumichrasti* | NT | 756 | Boback & Guyer 2003 |
| *Thamnophis valida* | NT | 1105 | Boback & Guyer 2003 |
| *Tomodon ocellatus* | NE | 460 | Boback & Guyer 2003 |
| *Tretanorhinus mocquardi* | NE | 765 | Kohler 2003 |
| *Tretanorhinus nigroluteus* | NE | 885 | Boback & Guyer 2003 |
| *Trimetopon barbouri* | NE | 260 | Kohler 2003 |
| *Trimetopon gracile* | NE | 300 | Kohler 2003 |
| *Trimetopon pliolepis* | NE | 287 | Kohler 2003 |
| *Trimetopon simile* | NE | 176 | Boback & Guyer 2003 |
| *Trimetopon slevini* | NT | 294 | Boback & Guyer 2003 |
| *Trimetopon viquezi* | NE | 250 | Kohler 2003 |
| *Trimorphodon biscutatus* | NE | 1750 | Kohler 2003 |
| *Tropidoclonion lineatum* | NT | 540 | Boback & Guyer 2003 |
| *Tropidodipsas fasciata* | NE | 742.5 | Boback & Guyer 2003 |
| *Tropidodipsas fischeri* | NE | 652 | Kohler 2003 |
| *Tropidodipsas sartorii* | NE | 857 | Kohler 2003 |
| *Tropidodryas serra* | NT | 1200 | Freitas & Silva 2005 |
| *Tropidodryas striaticeps* | NE | 1200 | Freitas & Silva 2005 |
| *Uromacerina ricardinii* | NE | 1000 | Freitas & Silva 2005 |
| *Urotheca decipiens* | NE | 569 | Boback & Guyer 2003 |
| *Urotheca fulviceps* | NE | 649 | Boback & Guyer 2003 |
| *Urotheca guentheri* | NT | 670 | Boback & Guyer 2003 |
| *Urotheca multilineata* | NE | 500 | Boback & Guyer 2003 |
| *Urotheca myersi* | NE | 349 | Boback & Guyer 2003 |
| *Urotheca pachyura* | NE | 725 | Kohler 2003 |
| *Virginia striatula* | NT | 320 | Boback & Guyer 2003 |
| *Virginia valeriae* | NT | 390 | Boback & Guyer 2003 |
| *Xenodon dorbignyi* | NE | 560 | Boback & Guyer 2003 |
| *Xenodon merremi* | NE | 1350 | Freitas & Silva 2005 |
| *Xenodon nattereri* | NE | 600 | Freitas & Silva 2007 |
| *Xenodon neuwiedii* | NT | 1000 | Freitas & Silva 2005 |
| *Xenodon rabdocephalus* | NE | 1000 | Freitas & Silva 2005 |
| *Xenodon semicinctus* | NE | 660 | Boback & Guyer 2003 |
| *Xenodon severus* | NE | 1212 | Boback & Guyer 2003 |
| *Xenopholis scalaris* | NT | 400 | Freitas & Silva 2005 |
| *Xenoxybelis argenteus* | NE | 1335 | Boback & Guyer 2003 |

**Source references**

1. Boback SM, Guyer C (2003) Empirical evidence for an optimal body size in snakes. Evolution 57: 345-451.
2. Campbell JA, Camarillo R JL, Ustach PC (1995) Redescription and rediagnosis of *Tantilla shawi* (Serpentes: Colubridae) from the Sierra Madre Oriental of Mexico. The Southwestern Naturalist: 120-123.
3. Canseco-Márquez L, Mendelson III JR, Gutiérrez-Mayén G (2002) A new species of large Tantilla (Squamata: Colubridae) from the Sierra Madre oriental of Puebla, Mexico. Herpetologica 58: 492-497.
4. Cope ED (1879) Eleventh contribution to the herpetology of tropical America. Proc Am Philos Soc: 261-277.
5. Daltry JC, Bloxam Q, Cooper G, Day ML, Hartley J, et al. (2001) Five years of conserving the ‘world’s rarest snake’, the Antiguan racer *Alsophis antiguae*. Oryx 35: 119-127.
6. Franco FL, de Carvalho Cintra LA, de Lema T (2006) A new species of Calamodontophis amaral, 1963 (Serpentes, Colubridae, Xenodontinae) from southern Brazil. S Am J Herpetol 1: 218-226.
7. Freitas MA, Silva TFS (2005) A herpetofauna da mata atlântica nordestina: Guia ilustrado. Pelotas:USEB.
8. Freitas MA, Silva TFS (2007) Guia ilustrado: A herpetofauna das caatingas e áreas de altitude do nordeste brasileiro. Pelotas: USEB.
9. Köhler G (2003) Reptiles of central america. Offenbach (Herpeton-Verlag).
10. Marques OA, Martins M, Sazima I (2009) A new insular species of pitviper from Brazil, with comments on evolutionary biology and conservation of the *Bothrops jararaca* group (Serpentes, Viperidae).
11. Passos P, Arredondo JC, Fernandes R, Lynch JD (2009) Three new Atractus (Serpentes: Dipsadidae) from the Andes of Colombia. Journal Information 2009.
12. Rossman DA, Wallach V (1987) *Adelophis Duges*. Mountain meadow snakes. Catalogue of American Amphibians and Reptiles, 408: 1-2.
13. Roth-Monzón AJ, Mendoza-Hernández AA, Flores-Villela OA (2011) *Lampropeltis ruthveni* (Serpentes: Colubridae) from the State of Hidalgo, Mexico. The Southwestern Naturalist 56: 430-431.
14. Terribile LC, Diniz‐Filho JAF, Rodríguez MÁ, Rangel TFL (2009) Richness patterns, species distributions and the principle of extreme deconstruction. Global Ecol Biogeogr 18: 123-136.
